# Supplementary material for: WFA-GPU: gap-affine pairwise read-alignment using GPUs
Source: Bioinformatics. 2023 Nov 17;39(12):btad701. doi: 10.1093/bioinformatics/btad701 (PMC10697739; doi:10.1093/bioinformatics/btad701)
Supplement: btad701_Supplementary_Data [file btad701_supplementary_data.pdf]

Supplementary material for WFA-GPU: Gap-affine pairwise read-alignment using GPUs

Quim Aguado-Puig, Max Doblas, Christos Matzoros, Antonio Espinosa, Juan Carlos Moure, Santiago Marco-Sola, and Miquel Moreto

S1 Experimental Datasets

Dataset	Sample	No. pairs	Seq. Length (bps) min	avg	max
Illumina 150	HG002[†]	100M	134	148	162
Illumina 250	HG002[†]	100M	140	248	275
PacBio CSS	HG002[†]	10M	263	9561	18317
PacBio HiFi	HG002[§]	10M	201	12847	24640
Nanopore	ERR3279200<br>ERR3279201	10M	104	6249	21708

Table S1: Description of the real datasets used in the experimental evaluation. [†]Datasets obtained from NIST's Genome in a Bottle (GIAB) project can be found at `https://github.com/genome-in-a-bottle/giab_data_indexes`. [§]Datasets obtained from PrecisionFDA Truth Challenge V2 can be found at `https://precision.fda.gov/challenges/10`.

S2 Experimental Evaluation on Simulated Data

		100 Million alignments × 150bps						10 Million alignments × 1Kbps						100K alignments × 10Kbps					
		e=2%		e=5%		e=10%		e=2%		e=5%		e=10%		e=2%		e=5%		e=10%	
		T(s)	R(%)	T(s)	R(%)	T(s)	R(%)	T(s)	R(%)	T(s)	R(%)	T(s)	R(%)	T(s)	R(%)	T(s)	R(%)	T(s)	R(%)
GPU	GASAL2	13	98.3	14	95.8	14	93.0	44	84.6	44	64.7	45	52.4	3219	47.0	3224	36.8	3229	34.1
	ADEPT	161	n/c	161	n/c	161	n/c	error	n/a	error	n/a	error	n/a	error	n/a	error	n/a	error	n/a
	NVBio	6	58.2	6	6.1	6	0.0	error	n/a	error	n/a	error	n/a	error	n/a	error	n/a	error	n/a
	CudaAligner†	113	95.3	113	66.1	117	25.7	86	9.1	86	1.6	86	0.3	20	0.9	20	0.0	20	0.0
	WFA-GPU	11	100	18	100	27	100	6	100	20	100	78	100	4	100	21	100	71	100
	├approx	11	100	18	100	27	100	6	100	11	100	21	100	1	100	2	100	4	100
	└distance	6	100	8	100	13	100	3	100	9	100	32	100	2	100	6	100	16	100
	└approx	6	100	7	100	10	100	3	100	4	100	7	100	1	100	1	100	2	100
CPU	Seqan	2028	100	2028	100	2190	100	9066	100	9288	100	9774	100	9912	100	10098	100	10440	100
	Parasail(strip)	8436	100	8358	100	8238	100	32274	100	32178	100	31842	100	33408	100	33306	100	32760	100
	Parasail(scan)	1197	100	1183	100	1197	100	3882	100	3902	100	3911	100	4869	100	4857	100	4825	100
	Parasail(diag)	1908	100	1914	100	1916	100	7482	100	7488	100	7488	100	7686	100	7692	100	7698	100
	Edlib†	210	95.9	202	69.6	200	30.0	118	67.6	118	9.5	135	0.0	36	1.8	38	0.0	43	0.0
	KSW2	298	100	306	100	317	100	876	100	882	100	882	100	2028	100	2034	100	2040	100
	WFA	12	100	37	100	78	100	15	100	61	100	209	100	14	100	83	100	272	100

Table S2: Time (T, in seconds) and recall (R, as a percentage of exact alignments) for simulated datasets with different error rates (e). All CPU executions use 10 threads. †Implementations can only produce edit-distance alignments.

Sequence length	150	1000	1000	10000	10000
Error rate (%)	5	5	10	5	10
WFA (CPU)	134	149	140	129	129
WFA-GPU	130	185	290	298	312

Table S3: Average power usage (in Watts) to compute alignments using the CPU and GPU aligners. CPU consumption is obtained using Linux perf and GPU consumption is obtained using nvidia-smi.

Sequence length	150	1000	1000	10000	10000
Error rate (%)	5	5	10	5	10
WFA (CPU)	1.4	2.5	8.1	3.0	9.8
WFA-GPU	0.6	1.0	5.1	1.9	6.1

Table S4: Power consumption (in Watt-hours) to compute alignments using the CPU and GPU aligners.

S3 Power consumption analysis

In this section, we analyze the power consumption enhancements derived from using WFA-GPU over WFA (CPU). It's worth noting that making an entirely equitable comparison is a challenging endeavor, as our analysis is tailored to our specific CPU-GPU configuration. We have tried to make the comparison as fair as possible, using a balanced CPU-GPU combination, however, we acknowledge that comparing diverse hardware devices introduces complexities in the evaluation process.

The thermal design power (TDP) of the NVIDIA GeForce 3080 is 320W, while the TDP of the Intel Xeon-W2155 is 140W. However, many hardware components, like CPU memory and the motherboard, are not accounted for in the CPU TDP. Moreover, hardware devices do not always work at their maximum TDP. As a reference, Table S3 presents the average power by the CPU and GPU when executing WFA (CPU) and WFA-GPU, respectively.

Table S3 shows that the GPU has a higher power draw during the alignment process, however, it is important to emphasize that WFA-GPU significantly reduces execution time, which makes WFA-GPU experiments more power-efficient in terms of energy consumption (measured in Watt-hours, or *Wh*) compared with CPU executions. Table S4 provides a comparison of power consumption (*Wh*) between CPU and GPU executions. Remarkably, our results demonstrate that WFA-GPU experiments consume less overall energy, even though GPU devices require more power than typical CPUs.

S4 Performance scalability

Our method is not constrained by the length of the input sequences, but rather by the sequence error rate (i.e., maximum alignment score). According to the maximum alignment score, WFA-GPU allocates the necessary memory, maximizing the total number of GPU workers spawned on the device. Ultimately, this allocation depends on the available computational resources of the device (i.e., Memory and Streaming Multiprocessors, SMs). Note that the memory taken by the input sequences is negligible compared to the memory required by the internal WFA structures. In Figure S1, we show the maximum number of GPU workers that can be accommodated on various GPU models, based on the maximum alignment score allowed.

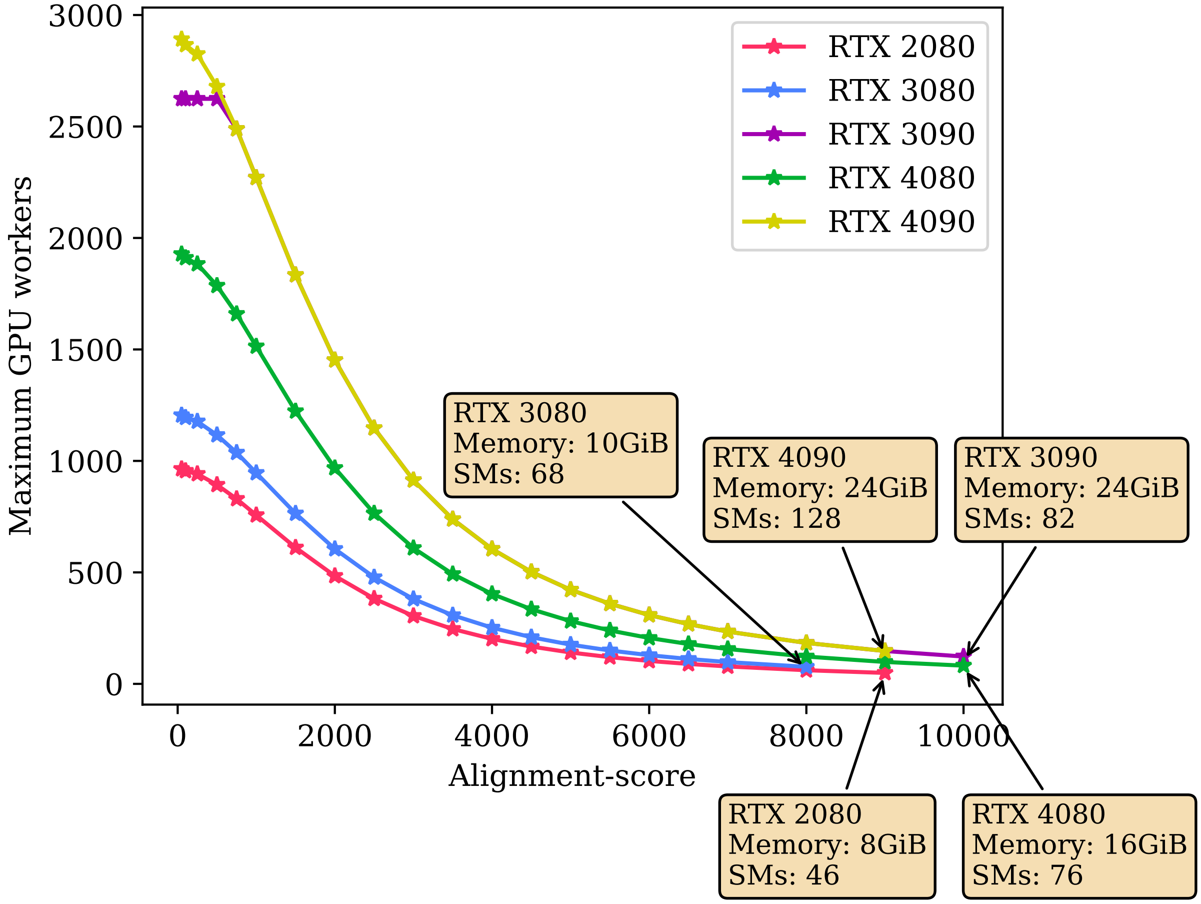

Figure S1: Maximum number of GPU workers that can be executed in parallel depending on the maximum alignment score for different GPU device models (i.e., different number of SMs and Memory).

Figure S1 shows how the maximum number of parallel GPU workers decreases as the maximum alignment-score increases. For large alignment-score values, only one GPU worker can be allocated per SM. Labeled points at the right of Figure S1 show the maximum alignment-score for different GPU models. For instance, an RTX 4080 GPU, equipped with 16GiB, could align a pair of sequences with a maximum alignment-score of 10,000.

Note that the RTX 4090, despite having more multiprocessors available, may not be able to handle alignments with alignment-scores as high as the RTX 3080. Although both have the same memory available, the former can execute more alignments in parallel, necessitating a larger memory footprint.

S5 Approximated WFA-GPU parameter exploration

The heuristic parameters affect execution time and recall (percentage of exact alignments). By changing width of the band (β) and the number of steps between the band re-centering (λ) we can find the balance point between time and recall.

It is essential to emphasize that utilizing a narrower band width (β) can significantly influence memory usage. Since employing more threads than the band size is impractical, opting for a smaller β leads to the creation of GPU workers with fewer threads. Consequently, this allows for accommodating a higher number of workers within each Streaming Multiprocessor (SM). This increased parallelism, however, comes at the cost of utilizing more GPU memory, as each worker necessitates its own dedicated memory space. It may be the case where using small bands for alignments with high errors generates a number of GPU workers that exceed the available memory capacity.

We present the Nanopore dataset as a case study, this dataset is the one that benefits the most from the adaptive strategy due to its long sequences and high error rates. Figure S2 shows how time and recall are affected by each (β, λ) combination. It can be seen that there is nearly no performance benefit on incrementing λ beyond 100. When using a large width for the band (β), a $\lambda = 100$ is sufficient and, when using a smaller β, setting λ around 50 is recommended. In any case, the accuracy drop is usually small ($< 3\%$). Therefore, unless optimal accuracy is paramount, default (β, λ) parameters will yield a good balance between execution time and minimal loss in recall.

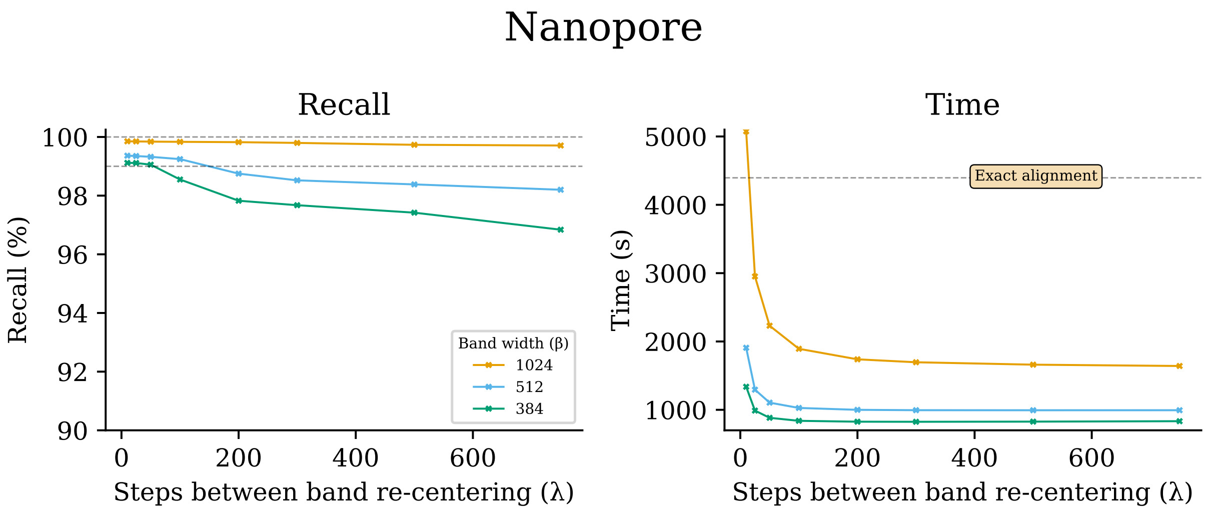

Figure S2: Recall (as a percentage of exact alignments) and time (in seconds) when aligning the Nanopore dataset using different β and λ parameters.
